# An Inexpensive 6D Motion Tracking System for Posturography

**DOI:** 10.3389/fneur.2018.00507

**Published:** 2018-06-29

**Authors:** William V. C. Figtree, Americo A. Migliaccio

**Affiliations:** ^1^Balance and Vision Laboratory, Neuroscience Research Australia, Sydney, NSW, Australia; ^2^Graduate School of Biomedical Engineering, University of New South Wales, Sydney, NSW, Australia; ^3^Department of Otolaryngology-Head and Neck Surgery, Johns Hopkins University, Baltimore, MD, United States

**Keywords:** posturography, sway, stereo vision, center-of-pressure, center-of-mass

## Abstract

Computerized posturography is most often performed with a force plate measuring center-of-pressure (COP). COP is related to postural control actions but does not monitor the outcome of those actions, i.e., center-of-mass (COM) stability. For a more complete analysis of postural control COM should also be measured; however, existing motion tracking technology is prohibitively expensive and overcomplicated for routine use. The objective of this work was to create and validate an inexpensive and convenient stereo vision system which measured a trunk-fixed target's 3D position and orientation relating to COM. The stereo vision system would be complementary to typical force plate methods providing precise 6D position measurements under laboratory conditions. The developed system's measurement accuracy was worst in the inferior-superior axis (depth) and pitch coordinates with accuracy measures 1.1 mm and 0.8°, respectively. The system's precision was worst in the depth and roll coordinates with values 0.1 mm and 0.15°, respectively. Computer modeling successfully predicted this precision with 11.3% mean error. Correlation between *in vivo* target position (TP) and COP was above 0.73 with COP generally demonstrating larger excursions oscillating around TP. Power spectral analysis of TP revealed 99% of the signal was bound below 1.1 Hz matching expectations for COM. The new complementary measurement method enables identification of postural control strategies and as a result more complete analysis. Stereo vision is a useful complement to typical force plate equipment. The system presented here is inexpensive and convenient demonstrating potential for routine use in clinic and research. In order to use this system in clinic, future work is required in interpretation of this system's data and normal reference values must be established across gender and age in a healthy population followed by values from patients with different pathologies.

## Introduction

The assessment of human postural control is an important outcome in clinic and research for evaluation of falls risk and identification of specific balance disorders (1). During quiet standing tasks, maintenance of a stable center-of-mass (COM) within a limit of stability is achieved by shifting the body's center-of-pressure (COP) based on multisensory input from visual, vestibular, and proprioceptive systems among others (2, 3). While there are mechanisms within the central nervous system to compensate for irregular sensory function, well-established methodology, such as the Romberg test, can identify abnormal systems by stressing the postural control systems (4). Thus, posturography can be used to differentiate the contribution of the visual, vestibular, and proprioceptive systems to a patient's balance. Posturography when coupled with a platform with apliable (e.g., foam) surface or a moveable platform can provide a functional test of vestibular function alone. For example, when the platform has a foam surface or sways to take away proprioception and the subject has their eyes closed, or when the platform sways and the visual surround moves with it so that proprioception and visual cues should be ignored. Although posturography cannot be used to localize a vestibular lesion there is a correlation between COP/sway and gaze velocity (a measure of visual stability mediated by the vestibulo-ocular reflex), i.e., COP/sway and gaze velocity is greater in patients with vestibular hypofunction compared to control subjects (5–7). Also, studies measuring gaze stability during balance perturbations delivered directly to the body show an inverse correlation between gaze fixation (larger is better) and latency to step (shorter is better, implying better postural stability) (8).

Computerization of these posturographic tests became prominent in the 1980s and employed a variety of technologies including: EMG, force plates, potentiometers, computer vision, wearable inertial sensors (accelerometers and gyroscopes), and electromagnetic trackers (1, 4, 9). To date postural control is most often evaluated using force plate measured COP as this methodology is sensitive to small changes in the subject's ability to balance, produces real-time results, and is both inexpensive and convenient (4, 10, 11). COP is a 2D variable, related to ankle torque, which provides insight into the subject's postural control mechanisms; however, it does not directly measure COM stability, the actual outcome of those mechanisms (12). While there are many successful methods for estimation of COM based on COP they are not widely adopted as they can be prone to error (10). It would instead be preferable to have a direct measure of sway trajectory complementary to COP which is convenient for measurement in routine practice.

The goal of this work is to create a system which captures the complete 6D motion of a body for posturographic testing. This system should be both inexpensive and convenient to implement and use. Stereo vision systems offer an appropriate solution; in fact, they are already well established in the study of gait and posture (13–16). However, their implementation often suffers from a lack of specialization, instead making use of expensive, one-size-fits-all commercial systems which need to be customized. They also frequently require the placement of many markers on the body which is time consuming and not ideal for routine use (4). There is a huge variety of affordable camera technology and code libraries specifically for calibration and implementation of computer stereo vision (17). In this work we take advantage of these resources to implement and validate a stereo vision system specifically for static posturography which is easy to use and, when used in complement to force plate measures, provides a more complete analysis of postural control. We present this implementation step-by-step from theoretical foundations to equipment validation. To date we are unaware of any other publication which covers these topics in such detail for static posturography.

## Materials and methods

### Theory

We developed a stereo vision system able to track a rigid body's 6D motion. The rigid body (or target) comprises three markers. Two cameras whose relative position and orientation are known, observe the same marker. By locating the marker's centroid in each 2D image plane the marker's 3D position centroid may be calculated relative to a predefined coordinate system. By tracking the three markers attached to the rigid body, the body's 6D position and orientation may be calculated by determining the linear transform which aligns the paired marker centroids from one frame to the next.

#### Marker centroid calculation

We begin with a classic camera model which maps a 3D point onto a 2D image plane using homogeneous coordinates. Homogenous coordinates allow operations such as rotation, translation, and perspective projection to be combined into a single matrix multiply operation (18); their use here greatly simplifies the mathematics involved. The mapping between ℝ^3^ Cartesian coordinates and ℝ^4^ homogenous coordinates is [x y z]↔[x⋅ wy ⋅ wz ⋅ ww] where *w* is a scaling factor. The classic pinhole camera model follows:

(1) $$\begin{array}{l} \left[\begin{array}{c} u\\ v\\ 1 \end{array}\right]=C\cdot E\cdot w\cdot \left[\begin{array}{c} x\\ y\\ \begin{array}{c} z\\ 1 \end{array} \end{array}\right] \end{array}$$

Where:

(2) $$\begin{array}{l} E=\;\left[R\;|\;T\right] \end{array}$$

(3) $$\begin{array}{l} C=\;\left[\begin{array}{c} \begin{array}{c} f_{u}\\ 0\\ 0 \end{array}\;\begin{array}{c} s_{k}\\ f_{v}\\ 0 \end{array}\;\begin{array}{c} p_{u}\\ p_{v}\\ 1 \end{array} \end{array}\right] \end{array}$$

Cartesian coordinates: *x, y*, and *z* are mapped to image plane coordinates: *u* and *v* by first transforming them to homogeneous coordinates with scaling factor *w*. Next the camera's extrinsic matrix *E* brings the coordinate frame to that of the camera's point of view, requiring the augmentation of a rotation (matrix *R*) and a translation (vector *T*) (19). Finally the camera's intrinsic matrix *C* projects the marker's homogeneous coordinates onto the camera's image plane. *C*'s elements are: horizontal and vertical focal length *f*_*u*_ and *f*_*v*_, respectively; horizontal and vertical principal point (focal center) coordinates *p*_*u*_ and *p*_*v*_, respectively; and camera skewness *s*_*k*_ (19).

A stereo vision system uses two such camera models observing the same 3D point, for instance, a marker centroid. Assuming the system is calibrated each camera's intrinsic and extrinsic matrix is known leaving a system of six equations with five unknowns, solvable for the observed centroid's coordinates.

A typical solution applies image rectification, a process which reprojects captured images onto a common image plane. As part of this process virtual camera models are defined such that: intrinsic skewness is zero; both horizontal and vertical focal lengths are equal and the same across cameras (*f*); the cameras' principle axes are aligned; and, assuming a horizontal stereo configuration, their optical center is offset only horizontally, i.e., along the baseline (*T*_*x*_). Operating in this new, virtual image plane, the camera models may be written as follows. Numeric subscripts 1 and 2 designate the left and right camera, respectively; the left camera is used as reference.

(4) $$\begin{array}{l} \left[\begin{array}{c} u_{1}\\ v_{1}\\ 1 \end{array}\right]=\left[\begin{array}{cccc} f & 0 & p_{u1} & 0\\ 0 & f & p_{v} & 0\\ 0 & 0 & 1 & 0 \end{array}\right]\cdot \left[\begin{array}{c} x\\ y\\ \begin{array}{c} z\\ 1 \end{array} \end{array}\right] \end{array}$$

(5) $$\begin{array}{l} \left[\begin{array}{c} u_{2}\\ v_{2}\\ 1 \end{array}\right]=\left[\begin{array}{cccc} f & 0 & p_{u2} & T_{x}\cdot f\\ 0 & f & p_{v} & 0\\ 0 & 0 & 1 & 0 \end{array}\right]\cdot \left[\begin{array}{c} x\\ y\\ \begin{array}{c} z\\ 1 \end{array} \end{array}\right] \end{array}$$

The solution to Equations (4) and (5) for centroid position (*x, y, z*) is Equation (6) below where horizontal disparity (*u*_2_ − *u*_1_) is mapped to the homogeneous coordinates of the viewed 3D position by reprojection matrix (or Q-matrix) *Q* (20).

(6) $$\begin{array}{l} w\left[\begin{array}{c} x\\ y\\ \begin{array}{c} z\\ 1 \end{array} \end{array}\right]=Q\cdot \left[\begin{array}{c} u_{1}\\ v_{1}\\ \begin{array}{c} {u_{2}-u}_{1}\\ 1 \end{array} \end{array}\right] \end{array}$$

Where:

(7) $$\begin{array}{l} Q=\left[\begin{array}{cccc} 1 & 0 & 0 & -p_{u1}\\ 0 & 1 & 0 & -p_{v}\\ 0 & 0 & 0 & f\\ 0 & 0 & -\frac{1}{T_{x}} & \frac{p_{u1}-p_{u2}}{T_{x}} \end{array}\right] \end{array}$$

Application of Equation (6) requires a calibrated system whose image rectification transforms and Q-matrix are known. In practice this information is gathered in a single calibration process where the stereo vision system is presented with multiple views of a known calibration pattern, typically a checkerboard or grid of dots. Since the pattern geometry (planar pattern, grid interval, size, etc.) is known a robust solution for each camera's intrinsic and extrinsic matrices as well as any non-linear image distortion can be estimated from the captured images. There are multiple algorithms available which perform this estimation, a good summary of which is provided by Dubrofsky (21). From this individual camera information, rectification transforms, and their corresponding Q-matrix can be calculated (20).

#### Target position and orientation estimation

**Figure 1:**
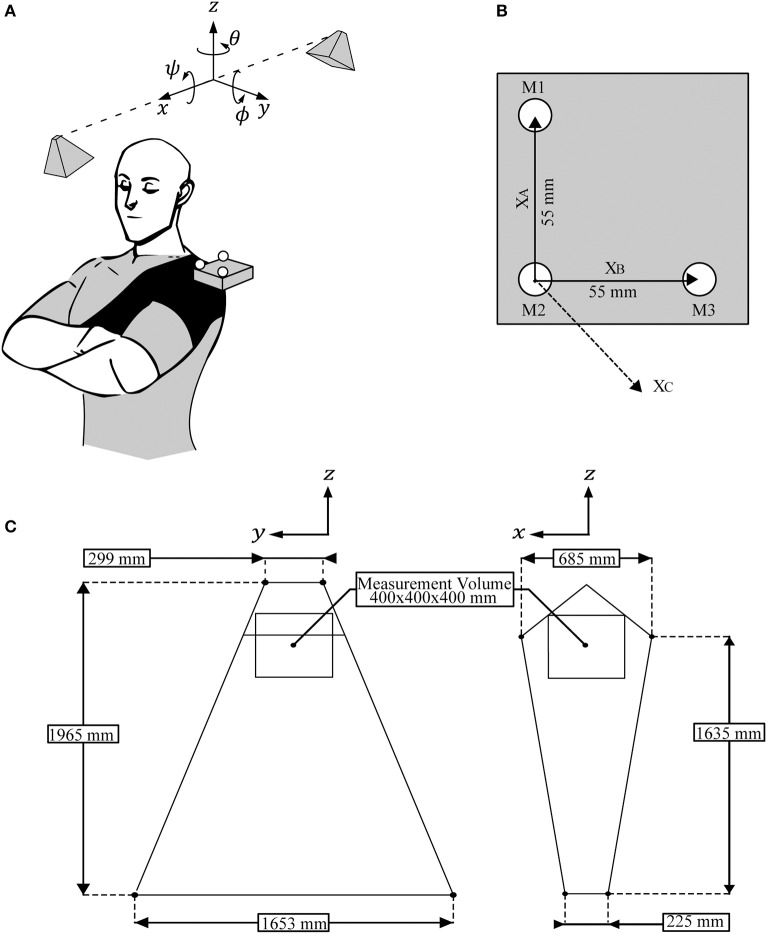
System implementation. **(A)** A subject wearing a shoulder brace with incorporated stereo vision target attached by a stiff pivot joint. The system's two cameras are mounted above the subject's left shoulder. The coordinate frame is aligned such that: the *z* axis is vertical, the *x* axis is horizontal and aligned with the stereo vision baseline, and the *y* axis is perpendicular to *x* and *z*. The subject is positioned with their anterior, left lateral, and superior directions aligned with the *x, y*, and *z* axes, respectively. **(B)** Top view of the stereo vision target. Markers M1, M2, and M3 are affixed in an L pattern on the target's top surface with 55 mm spacing. Vector *X*_*A*_ is the difference between the position of M2 and M1. Similarly vector *X*_*B*_ is the difference between the position of M2 and M3. Vector *X*_*C*_ is the cross product of *X*_*A*_ and *X*_*B*_. **(C)** The volume viewable by both of the system's cameras in the *yz* plane (left diagram) and the *xz* plane (right diagram). Posturographic measurements are taken from the labeled measurement volume which is a cube with 400 mm sides aligned with the system's axes and centered at a height of 1,370 mm. It is a sub-volume of the viewable volume.

A minimum of three non-collinear points are required to determine the orientation of a 3D rigid body. The change in position of these three points from one frame to the next is used to calculate the change in both position and orientation of the rigid body. We use a rigid body consisting of an L shaped marker pattern where a marker is placed in the bend (M2) and at each end point (M1 and M3) of the L (see Figure 1B).

We define target position (TP) as the translation of marker 2 from an initial reference frame to the current frame:

(8) $$\begin{array}{l} P=M_{2}-\;M_{2ref} \end{array}$$

In order to calculate the orientation of the target a minimum of three non-planar vectors are required to form a basis. As the rigid body rotates, so does the basis. We defined the basis vectors:

*X*_*A*_, Marker 1's position vector subtracted by Marker 2's position vector;

*X*_*B*_, Marker 3's position vector subtracted by Marker 2's position vector; and

*X*_*C*_ = *X*_*A*_ × *X*_*B*_.

By defining marker 2 as the origin we isolate the rotational component of the motion.

The orientation of the object can then simply be defined as the rotation matrix which rotates the initial basis to the current basis (22):

(9) $$\begin{array}{l} R=\left[\begin{array}{c} X_{A}\;X_{B}\;X_{C} \end{array}\right]\cdot {\left[\begin{array}{c} X_{A}\;X_{B}\;X_{C} \end{array}\right]}_{ref}^{-1} \end{array}$$

We decompose this rotation into a set of Fick Euler angles (rotation sequence: roll—ψ, pitch—ϕ, and yaw—θ) using the following conversion formula where numeric subscripts designate individual elements of rotation matrix *R* (23).

(10) $$\begin{array}{l} \left[\begin{array}{c} \psi \\ \phi \\ \theta \end{array}\right]=\;\left[\begin{array}{c} atan2\left(\frac{R_{32}}{\cos\left(-\mathrm{asin}\left(R_{31}\right)\right)}\;,\;\frac{R_{33}}{\cos\left(-\mathrm{asin}\left(R_{31}\right)\right)}\right)\\ -\mathrm{asin}\left(R_{31}\right)\\ atan2\left(\frac{R_{21}}{\cos\left(-\mathrm{asin}\left(R_{31}\right)\right)}\;,\;\frac{R_{11}}{\cos\left(-\mathrm{asin}\left(R_{31}\right)\right)}\right) \end{array}\right] \end{array}$$

### Implementation

Our stereo vision system consists of two monochrome cameras (BFLY-PGE-13E4M-CS, PointGrey, Canada) externally triggered with a microcontroller (MK20DX256, NXP Semiconductors, Netherlands) to capture simultaneous 640 × 512 pixel resolution frames at 100 Hz. Each camera has a lens (12VM412ASIR, Tamron, Japan) adjusted to a focal length of 6.9 mm with an IR low pass filter (R5000212478-15188, Edmund Optics, USA) mounted externally. These cameras are ceiling mounted at a height of 2,275 mm with a baseline of 870 mm and a vergence angle of 54°. This configuration views the specified measurement volume, see Table 1 and Figure 1C.

**Table 1:** System specifications.

**Parameter**	**Specification**	**Source**
Bandwidth	0.01–10 Hz	Expected bandwidth of posturographic motion (24)
Sampling frequency	100 Hz	Recommended rate based on the degradation of typical posturographic parameters when subsampling signals (24)
Measurement volume	400 mm cube at 1,370 mm height	This cube encompasses the 95th male percentile and 5th female percentile shoulder height, 1,525 and 1,215 mm, respectively, and is centered between the two (25). The cube is wide enough to encompass the typical limit of stability (LOS), 196 mm, observed in young healthy male subjects [LOS = 1525 mm* tan(7.34°) = 196 mm] (26)
Position resolution	4.2 mm	This position resolution is equal to the standard deviation of the absolute values of the test-retest difference of COP range over 10 sessions and 10 subjects. Data is collected from healthy subjects standing on a solid surface (27). Such resolution should minimally impact measurement noise
Orientation Resolution	0.2°	This orientation resolution is calculated from the position resolution by assuming an inverted pendulum sway model and worst case (minimum) subject height equal to 1,215 mm (5th female percentile shoulder height). [Orientation Resolution = (180°* 4.2 mm)/(π* 1,215 mm) = 0.2°]

The target consists of a 3D printed, ABS plastic body which has a compartment for some minor electronics and a 55 mm L shaped pattern on top with recesses for each marker. A spherical marker is fixed at the bend and each endpoint of the L pattern (Figure 1B). These markers are white, semi-opaque, 12 mm diameter, Acetal plastic ball bearings (BL-01200-AC, Miniature Bearings Australia, Australia). Markers are backlit with IR LEDs (TSHF6410, 890 nm, Vishay Semiconductors, USA). This choice of spherical marker and backlighting produces bright circular disks in the image plane which are easily tracked and whose COM corresponds to the same 3D marker centroid in both image planes. The target is mounted on a stiff pivot joint and incorporated into a shoulder brace (538CP Shoulder Support, LP Support, USA). The brace is strapped firmly around the subject's upper arm and torso so that it moves rigidly with the subject, see Figure 1A. The pivot joint allows an operator to adjust the target to approximately face the stereo vision system.

Our stereo vision system is controlled through a custom user interface written using NI LabVIEW 2014 SP1 f3 and NI Vision Development Module 2014 SP1. Calibration was performed using LabVIEW's stereo calibration example program. The calibration pattern used was a flat grid of 15 × 12 black dots on a white background with 26.4 mm dot spacing and 13.5 mm dot diameter. This provided: intrinsic, C, and extrinsic, E, camera matrices as well as the reprojection matrix, Q, and image rectification transforms. Captured images were rectified as per calibration; such that Equation (6) could be used to calculate the position of marker centroids. Captured images were then low pass filtered (each pixel's intensity was set to the average of the surrounding eight pixels) to reduce the random noise arising from the image sensor electronics.

Markers were identified in each image by searching for their key defining features, specifically markers are: bright, of a certain size, round, and slow moving. After low pass filtering, images were intensity thresholded to keep only the brightest image segments; this separated the markers from their background and reduced the complexity of further image processing. The resulting, bright image segments were then filtered by their: area (number of pixels) keeping only those segments which were the expected size of a marker; and their Heywood circularity (28), keeping only those segments which were sufficiently round to be a marker. The remaining segments' centroids were then calculated using a COM algorithm (28). Finally, segments were identified as markers in one of two ways. First-time execution identified markers by looking for the target's known L-shaped pattern; an operator could repeat this first-time execution whenever they deemed the images to be suitable for such identification (i.e., when there were few artifacts or cluttering segments). Subsequent execution identified markers by finding the segments which would result in the smallest marker movement; each permutation of paired segments and last known marker centroids were compared and the permutation with the lowest cost (defined as the summed straight-line distances between current potential marker centroids and the known prior marker centroids) was selected.

Having identified the marker centroids in each image plane the 3D marker positions were calculated using Equation (6) and stored as the current basis. The reference basis was manually selected from some prior time. Given the reference basis and the current basis the target's position (Equation 8) and orientation (Equation 10) were calculated for each frame and saved to disk. Prior to analysis the target's position and orientation signals were low pass filtered with a 10 Hz, 10th order, zero-phase, Butterworth filter keeping the signal's bandwidth as per Table 1 and reducing high frequency noise.

### System modeling

The stereo vision system was developed and tested in a temporary environment. A key aspect of its development was modeling the expected measurement precision. Such modeling gave confidence in system performance prior to installation and in-place validation. All modeling was performed using MATLAB R2016b.

The law of error propagation maps the uncertainty of independent variables to the uncertainty of dependent variables (29). It forms the foundation of our modeling approach and can be written as follows for a linear system approximation (30).

(11) $$\begin{array}{l} \Lambda_{F}=J\left(\bar{x}\right)\;\cdot \;\Lambda_{x}\;\cdot \;J{\left(\bar{x}\right)}^{T} \end{array}$$

Here: Λ_*x*_ and Λ_*F*_ are the covariance matrices of independent variables and dependent variables, respectively; $\bar{x}$ is the system model defining the relationship between independent and dependent variables; and $J\left(\bar{x}\right)$ is the Jacobian of $\bar{x}$ with partial derivatives taken with respect to the independent variables.

Application of the model requires quantification of the uncertainty on the model's inputs, and repeated calculation of the expected uncertainty of the system's outputs given the variety of situations we reasonably expect. We performed modeling as a two-step process first simulating the precision of the measurement of a single marker's position (equivalent to the target's position) and then secondly simulating the precision of the measurement of the target's orientation.

When simulating position measurement the model ($\bar{x}$) is Equation (6). This has independent variables: *u*_1_, *u*_2_, and *v* comprising the centroids of a marker in rectified image space; and dependent variables: *x, y*, and *z*. The uncertainty in marker centroids is typically dominated by quantization uncertainty and in the absence of further details is often estimated as a standard deviation equal to 0.5 pixels (30). Other application specific estimates can be found in literature, for instance for fiducial localization (31, 32) or edge detection (33, 34). We bypassed such estimates and measured input uncertainty directly in order to produce a more accurate system model. We measured the centroid of one marker over 10 s at the closest, central, and furthest distances expected for our application (700, 900, and 1,100 mm, respectively). The average centroid variance was 0.001819 pixels^2^. Assuming no covariance between centroid coordinates we substituted this variance into Λ_*x*_ and determined an expression for Λ_*F*_ using MATLAB's symbolic expressions. This expression is dependent upon marker centroid coordinates which can be determined for any marker position using Equations (4) and (5). The estimated uncertainty of *x, y*, and *z* was then calculated for each marker position in a 3D grid matching the measurement volume (Table 1) with 100 mm spacing.

When simulating orientation measurement the model ($\bar{x}$) is Equation (10). This has nine independent variables: the *x*, *y*, and *z* coordinates of the three target markers; and dependent variables: ψ, ϕ, and θ. The expected uncertainty of the marker position coordinates can be substituted directly from the prior position measurement simulation. Assuming no covariance between these coordinates we substituted into Λ_*x*_ and determined an expression for Λ_*F*_ using MATLAB's symbolic expressions. The expression is dependent upon the 3D marker positions which can be determined by rotating a horizontal reference model matching the target's dimensions. The estimated uncertainty of ψ, ϕ, and θ was then calculated for each target orientation in a polar grid with range ±20° in each axis and 10° spacing.

### System validation

The goal of system validation is to provide accuracy and precision statistics regarding the measurement of TP and orientation. Although precision is easily quantified through repeated measures, accuracy can only be obtained by comparing measurements to a reference. We used equipment that manually controlled the position and orientation of the target to provide this reference. Horizontal TP was controlled using a grid with 1 mm increments. This grid was mounted on a vertical sliding axis for depth control. Target orientation was controlled using a manual 3D gimbal with 1° increments.

The accuracy, trueness, and precision of the system's measurement of TP were evaluated by translating the target using the sliding grid. The target was sequentially fixed at points in the grid pattern matching the system's measurement volume, a 400 mm cube centered along the baseline at a depth of 900 mm. Using 100 mm grid spacing 125 fixation points were defined. Each position was held for 1 s providing 100 observations per position. Prior to evaluation a reference target was measured at the center of the cube to define the coordinate frame from which relative translations were measured. This was orthogonalized using the Gram-Schmidt process and aligned to the coordinate frame with a slight rotation. Errors for each *xyz* component of position were defined for each observation as the difference between the grid location and measured marker position. The *xyz* accuracy of each observation was defined as the absolute value of the errors. The accuracy, trueness, and precision of each fixation point were then defined as the mean accuracy, mean error, and error standard deviation, respectively, of all observations corresponding to that fixation point. Finally the typical accuracy, trueness, and precision of any subset of the measurement volume were defined as the median accuracy, trueness, and precision of the fixation points within that subset. Subsets taken included: all fixations, to provide a measure of typical performance; and horizontal grid levels, to provide a performance trend with increasing depth.

The accuracy, trueness, and precision of the system's measurement of target orientation were evaluated by manually rotating the target using a gimbal. The target was fixed in a 3D polar grid pattern which spanned ±20° about each axis and used a spacing interval of 10°. The gimbal was translated vertically to the closest, central, and furthest depths in the measurement volume (700, 900, and 1,100 mm, respectively). Evaluating the polar grid pattern at each of these depths defined a total of 375 orientations. Each orientation was held for 1 s providing 100 observations per orientation. Prior to evaluation of each polar grid pattern a reference target orientation was defined from which relative orientations could be determined. This reference target was centered along the baseline and oriented such that its markers lay in a horizontal plane facing the system's cameras. For each observation the 3D rotation (difference rotation) between the measured orientation and the polar grid orientation was determined. Errors for each observation were defined as the Euler Fick angles of this difference rotation. The ψφθ accuracy of each observation was calculated as the absolute value of the errors. The accuracy, trueness, and precision of each orientation were calculated as the mean accuracy, mean error, and error standard deviation, respectively, of the 100 observations corresponding to each orientation. Finally the typical accuracy, trueness, and precision of any subset of these orientations were defined as the median accuracy, trueness, and precision of that subset. Subsets taken included: all orientations, to provide a measure of typical performance; all orientations at each depth, to provide a performance trend with increasing depth; and orientations pooled by rotation purely about each axis, to provide a performance trend with changing target orientation.

### *In vivo* validation

Participation in this study was voluntary and informed written consent was obtained as approved by the University of New South Wales Human Ethics Committee.

To provide insight into the benefits of a complementary stereo vision—force plate system an *in vivo* validation was performed. A custom z-axis force plate was used to capture vertical ground reaction forces at 100 Hz. The force plate consisted of a 450 × 450 mm steel plate supported by load cells in each corner (Xtran S1W 750N, Applied Measurement Australia, Australia). From these ground reaction forces the subject's instantaneous COP was calculated (35). COP was then filtered to match the stereo vision system with a low pass 10 Hz, 10th order, zero-phase, Butterworth filter.

One subject (male, age 69) with left sided superior vestibular neuritis (onset 8 months prior to assessment in this study), as confirmed by a Neurologist (clinical assessment upon referral included: Romberg test positive on foam, video head impulse test on horizontal canals [right canal gain = 0.76, left canal gain = 0.5 with a volley of overt refixation saccades 120–150 ms after head impulse onset], no observed spontaneous or positional nystagmus, no observed gait difficulty), was simultaneously recorded with the force plate and the stereo vision system. Recordings included capture of a shared external trigger which was used to synchronize each data time series. The subject stood on a foam surface (to limit proprioceptive input and increase the balance challenge) with their feet together and arms crossed for a period of 20 s under two conditions. The first condition required the subject's eyes to be open (predominantly visual and vestibular input) and the second required them to be closed (predominantly vestibular input).

Stereo vision was compared to COP by extracting the stereo vision target's *x* and *y* position data. System correlation was assessed using Spearman's correlation coefficient. In the time domain, data was assessed using traditional posturographic parameters: path length, the total distance traveled by a point; and the range of *x* and *y* position. Frequency content was assessed by computation of the power spectral density (PSD) of each signal by Welch's method followed by calculation of the frequency below which 99% of the power spectrum is contained (referred to as f99) (36).

## Results

### System performance

**Figure 2:**
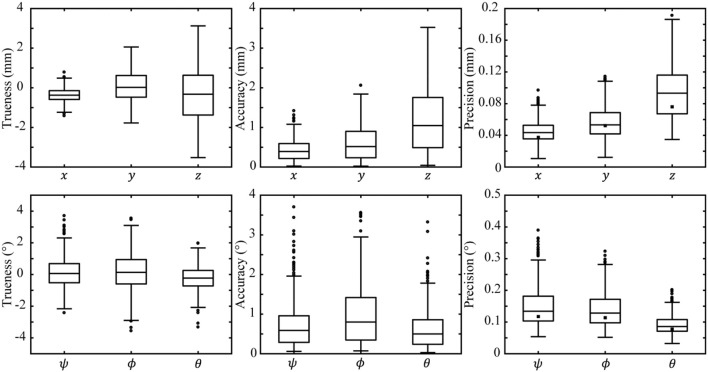
Results of validation and modeling evaluated over the whole measurement volume. The top and bottom rows plot measurement trueness (left column), accuracy (middle column), and precision (right column) for position and orientation, respectively. Validation data of each coordinate are represented as a box plot. The central line of each box plot indicates the median of its data, the top and bottom edges indicate the 1st and 3rd quartiles, respectively, and the whiskers represent the minimum and maximum datum excluding outliers. Outliers (datum further than 1.5 times the inter quartile range from the 1st and 3rd quartiles) are drawn as open circles. Median modeled precision is overlayed on each box plot as a filled square.

Typical performance was estimated by summary statistics of trueness, accuracy, and precision of position and orientation measures calculated from the complete 375 validated positions and orientations. Performance mean and standard deviation is presented in Table 2. Performance five number summaries (median, 1st, and 3rd quartiles, maxima, and minima) are presented as box plots in Figure 2. These box plots provide a detailed view of typical performance and the spread of that performance across the measurement volume. We use the median as a measure of performance rather than mean because it is more robust to outliers.

**Table 2:** Typical performance results of target position and orientation measurement from validation and modeling.

**Performance measure**	**Position (mm)**			**Orientation (°)**		
	***x***	***y***	***z***	**ψ**	**ϕ**	**θ**
Trueness	−0.3710 ± 0.3494	0.0682 ± 0.7404	−0.3483 ± 1.3883	0.1623 ± 1.2318	0.1412 ± 0.7750	−0.2342 ± 0.8711
Accuracy	0.4244 ± 0.2821	0.6017 ± 0.4361	1.1778 ± 0.8114	0.7418 ± 0.7657	0.9747 ± 0.5111	0.6277 ± 0.8711
Precision	0.0449 ± 0.0141	0.0567 ± 0.0206	0.0955 ± 0.0338	0.1492 ± 0.0559	0.1412 ± 0.0306	0.0918 ± 0.0566
Modeled precision	0.0384 ± 0.0062	0.0530 ± 0.0084	0.0779 ± 0.0239	0.1182 ± 0.0048	0.1155 ± 0.0030	0.0783 ± 0.0023

*Typical performance measures are presented as mean ± standard deviation data across all tested positions and orientations*.

Median measurement trueness is within 0.4 mm and 0.23° for each coordinate; however, a wide interquartile range demonstrates a large spread of measurement trueness across possible target positions and orientations. Measurement accuracy thus better demonstrates the typical error expected on a given datum with median accuracy <1.1 mm and <0.8° for each coordinate. Accuracy positive skew is due to its calculation as the absolute value of errors. Measurement precision is a good estimate of noise and possible measurement resolution, median precision is <0.10 mm and <0.14° for each coordinate.

System modeling gave an estimate of system performance prior to installation and validation. modeling was performed across 375 TP s and orientations matching the validation procedure and the measurement volume. Modeling results were summarized by: mean and standard deviation presented in Table 2; and median presented in Figure 2. The mean difference between modeled and validated precision was 11.3% with modeling always underestimating the validated result. The minimum 1.7%, and maximum 18.5%, differences occurred in the *y* and *z* coordinates, respectively.

### Effect of target depth

**Figure 3:**
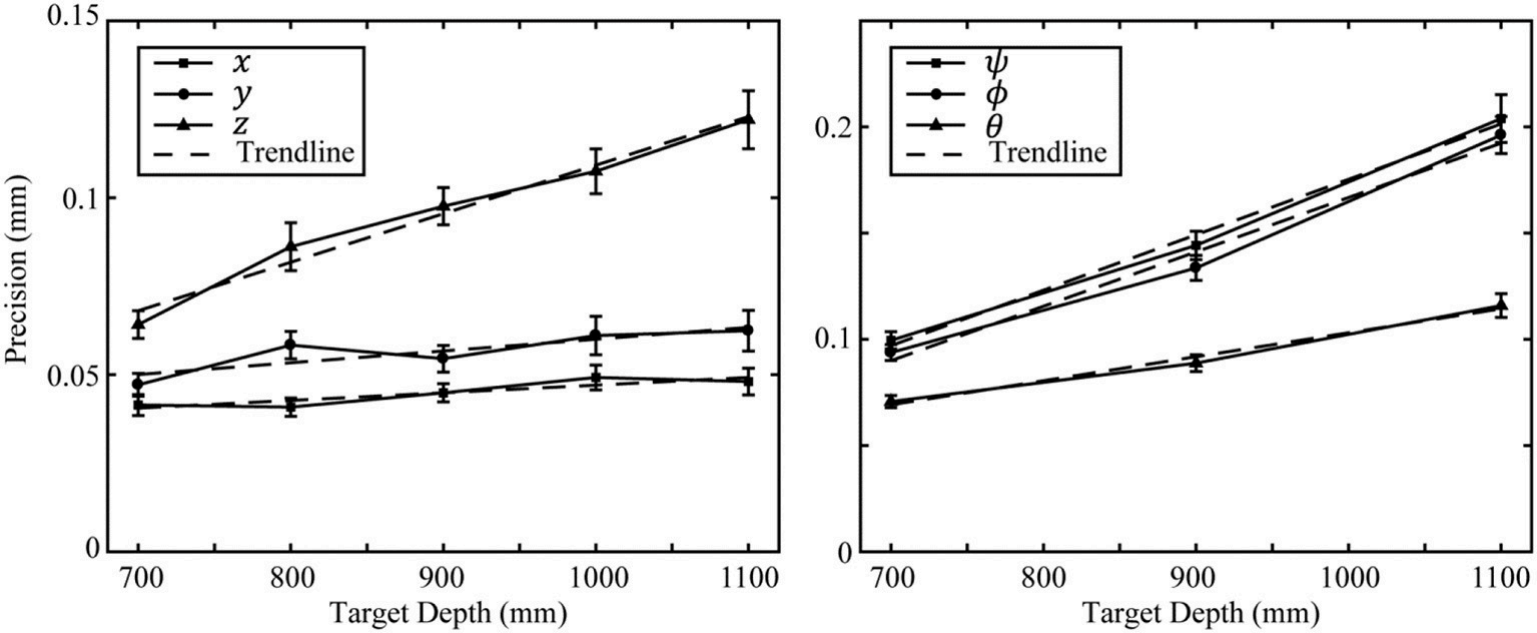
Precision depth trends. The left figure plots position measurement precision against target depth. The right figure plots orientation measurement precision against target depth. Error bars represent the 95% confidence interval of each datum. Linear trend lines are plotted for each data series.

The effect of target depth on measurement precision was investigated by calculating the mean and 95% confidence interval of precision data pooled by test depth. At each depth there were a total 75 positions and 125 orientations. Precision trends plotted against depth are presented in Figure 3. Measurement precision of *z*, ψ, ϕ, and θ demonstrated the strongest linear trends (R^2^ > 0.97) with precision worsening with increasing depth. The *z* coordinate had the most pronounced depth trend of the position coordinates with slope equal to 0.00014 mm/mm. Trends for *x* and *y* also had a strong linear fit (*R*^2^ = 0.82 and *R*^2^ = 0.735, respectively) but had near zero slope i.e., <0.00004 mm/mm. The ψ and ϕ coordinates have the most pronounced depth trend of the orientation coordinates with equal slope 0.00026°/mm. The θ coordinate is affected by depth approximately half as much as ψ and ϕ with slope equal to 0.00011°/mm.

### Effect of target orientation

**Figure 4:**
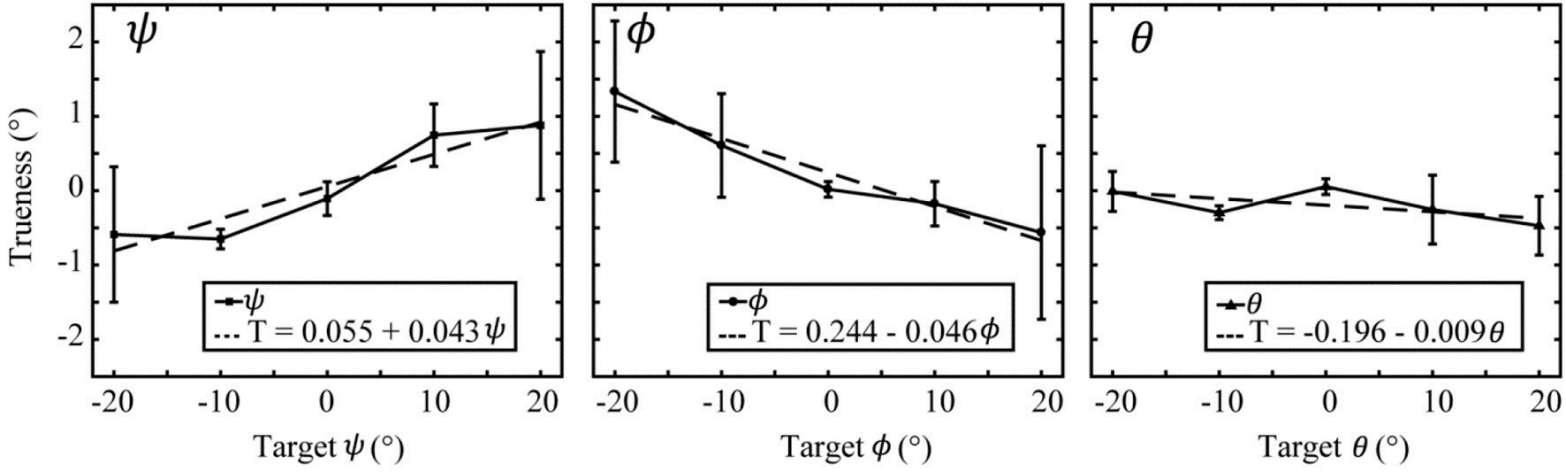
Orientation trueness plotted against target orientation. Pure rotations about the roll axis (left figure), pitch axis (middle figure), and yaw axis (right figure) are shown. Error bars represent the 95% confidence interval of each datum. Trend lines are plotted for the measurement trueness corresponding to each figure's primary axis.

The effect of target orientation on orientation measurement trueness was investigated by calculating the mean and 95% confidence interval of trueness data pooled by orientation displacement. Only target orientations due purely to rotation about a single axis were considered, other data was discarded. At each orientation a total of three measures contributed from each of the three tested depths. Orientation trueness trends are presented in Figure 4. For clarity, only the data regarding measurement of the changing coordinate is shown.

Changing ψ most affected the measurement trueness of ψ (*R*^2^ = 0.90, Trueness = 0.055 + 0.043ψ), ϕ and θ were comparatively unaffected (*R*^2^ < 0.52, slope < 0.010°/°). Changing ϕ most affected the measurement trueness of ϕ (*R*^2^ = 0.95, Trueness = 0.244–0.046ϕ); although, both ψ and θ were also affected (*R*^2^ = 0.79, and *R*^2^ = 0.58, respectively; slope < 0.03°/°). Changing θ did not significantly affect trueness in any coordinate, most affected was ϕ (*R*^2^ = 0.76, Trueness = −0.070–0.012θ), other coordinates exhibited almost no effect (*R*^2^ < 0.42, slope < 0.009°/°).

Orientation error measured as a percentage of actual target orientation can be extracted from the slopes of these linear regression fits. Regarding the data belonging to the changing coordinate, orientation error was: 4.3% in ψ, 4.6% in ϕ, and 0.9% in θ; although, the fit for θ is poor (R^2^ = 0.417).

### *In vivo* methods comparison

**Figure 5:**
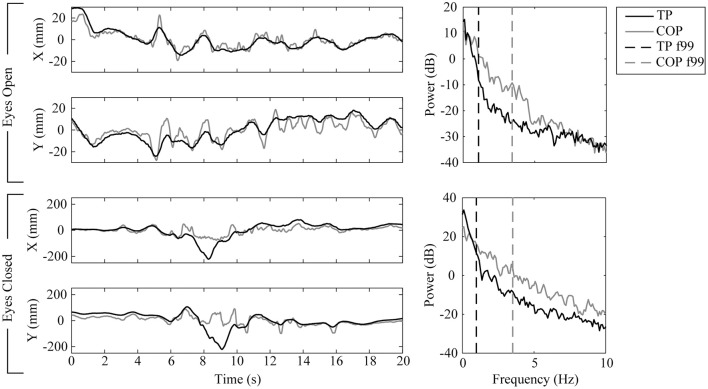
*In vivo* method results for one subject tested while standing on a foam surface under two conditions: eyes open (top half), and eyes closed (bottom half). Force plate recorded COP results and stereo vision recorded target position (TP) results are rendered in gray and black, respectively. Time series (left column) for each condition are split into *x* (top graph) and *y* (bottom graph) coordinates. Power spectrums (right column) estimate the distribution of power among the time series' frequency components; each time series' f99 parameter (the frequency below which 99% of the signal's power is contained) is overlayed as a vertical dashed line.

A single subject's force plate measured COP and stereo vision measured *x* and *y* (TP) were collected over 20 s while standing on a foam surface under two conditions: eyes open, and eyes closed (Figure 5). The two system's time series data were well correlated under both conditions (Spearman's, *p*_1_ = 0.87 and *p*_2_ = 0.73). COP contained a broader power spectrum (f99_1_ = 3.42 Hz, f99_2_ = 3.52 Hz) compared to TP (f99_1_ = 1.07 Hz, f99_2_ = 0.97 Hz). The broader power spectrum contributed to a longer path length in COP (path_1_ = 832 mm, path_2_ = 3,176 mm) compared to TP (path_1_ = 350 mm, path_2_ = 1,778 mm). Each system's position range was similar during the eyes open condition (range_COP_ = [43 mm, 47 mm], range_TP_ = [44 mm, 43 mm]) but differed significantly during the eyes closed condition (range_COP_ = [128 mm, 189 mm], range_TP_ = [303 mm, 327 mm]). There is a clear change in behavior during the 6.5 to 10 s interval in the eyes closed condition. Removing this interval, the eyes closed condition no longer shows such a significant difference between system's position range (range_COP_ = [109 mm, 149 mm], range_TP_ = [144 mm, 122 mm]). Both systems demonstrated an increase in task difficulty from the eyes open to the eyes closed condition with an order of magnitude increase in the path length and position range parameters.

The change in behavior during the 6.5–10 s interval reflects a change in balance strategy due to sudden imbalance. In the *x* (anterior-posterior) direction at 6.5 s the subject pressed hard with the front of their foot as shown by the COP data. This shifted their COM back such that COP was directed through the subject's heel, shown by the flat oscillation between 7.5 and 9 s in COP data. While the subject's COP was directed through their heel the subject's trunk continued backwards until they were able to regain postural control by pivoting forwards at their hips during the 8–9 s period as shown by the TP data. This backward and then forward trunk motion and inferred hip pivot response was captured by the complementary stereo vision—force plate system. It would have been missed had either of these systems not been present.

In the same period of instability a change in balance strategy is also observed in the *y* (mediolateral) direction. Ordinarily the subject's body acts approximately as an inverted pendulum since their feet are together. However, at 8 s the subject loads their left foot heavily, unloading their right foot (as seen in the leftwards COP data) and simultaneously shifts their shoulder rightwards (as seen in the TP data). As a result the subject acts as a double inverted pendulum and they are able to control their COM by applying torque both with their ankles and hips. Again this change in posture and control strategy would not have been captured without the complementary system.

## Discussion

The developed stereo vision system is able to measure target position and orientation within the specifications required for posturography. Position measures are, at worst, precise to 0.1 ± 0.04 mm and orientation measures are, at worst, precise to 0.15 ± 0.06°. However, there is systematic inaccuracy which, depending on the coordinate, typically contributes 0.4–1.1 mm position error and 0.5–0.8° orientation error. Modeling closely predicted system precision (mean error = 11.3%) but did not model systematic error. *In vivo* comparison between force plate measured COP and stereo vision measured *xy* TP demonstrates good correlation (Spearman's, ρ ≥ 0.73) in the time series data. Comparable *in vivo* parameters show similar changes between conditions for both systems. During periods of instability the complementary stereo vision—force plate system provided additional insight to the subject's posture and control.

We followed an affordable approach to stereo vision and in keeping with this methodology we used manual equipment for validation rather than expensive, highly accurate robotic (37), or vision systems (38). As such we expect that a significant portion of the quoted positional inaccuracy is contributed by the validation equipment and is not actually inherent to the stereo vision system. Equipment gradation creates an imperfect reference contributing an unpredictable bias to each measured error. The sliding grid's 1 mm position gradation and the gimbal's 1° orientation gradation are expected to have contributed up to 0.5 mm position error and 0.5° orientation error in each coordinate. Had a more accurate reference been used in validation remaining systematic error could have been corrected by a model. Nevertheless the reported inaccuracy is small as compared to force plate errors which have been reported up to ±30 mm (35). Therefore, we consider the affordable approach taken here to be suitable for this application.

The trend of position measurement precision worsening with increasing depth is a well-known phenomenon of stereo vision systems as is the fast rate of precision decline in the depth measurement itself (39). It follows that any orientation measure based on such position measurements would also have worsening precision due to error propagation and it is unsurprising that the orientation coordinates most dependent on a depth measure (ψ) and ϕ would be the most affected.

It is surprising that there is a strong relationship between target orientation error and target orientation. This effect is not significantly observed in pure θ rotations but contributes up to 5% error in the ψ and ϕ coordinates. This is due to the target design. The intention of the target's spherical markers was to produce circular image segments whose centroids corresponded to the markers' exact 3D centers. However, due to the beam pattern of the infra-red backlighting, it was observed that instead elliptical image segments were produced when the target was tilted away from each camera's optical axis. These elliptical segments became increasingly eccentric the further the target was tilted. This contributed error in two ways: first, given each camera had a separate point of view, their image segment centroids no longer corresponded to the same 3D location which produced a mismatch and violated the assumptions made for accurate position estimation; second, since this elliptical pattern changed with tilt, the same 3D point is not necessarily tracked between frames which resulted in under or overestimation of target movement. This suggests the more common passive target design utilizing reflective markers and global lighting would be more accurate at large target angles. However, external lighting can create complicated scenes in the presence of unanticipated reflective objects which increases measurement setup complexity and time. Our approach prioritizes easy setup and the observed backlighting effect could be minimized with an improved target design. Such a design would reduce the directionality of the LEDs and position them at the center of the spherical markers.

While considering system design it is worth exploring the possibility of including redundant markers on the target and also the trade-offs between the simple solution presented here and other more complex solutions for target position and orientation estimation. The approach presented here is by far the simplest, three markers is the minimum required for 3D orientation calculation, and a minimum of one marker (M2) is used to define 3D TP. However, this minimal approach does cause problems when markers are occluded; when any marker is obscured orientation information is lost, similarly if M2 is obscured position information is lost. Without altering hardware design a marginally more robust solution for TP may be defined as the mean marker position. Then given any combination of visible markers a TP is defined. Unfortunately the mean position changes depending on the set of visible markers creating discontinuities and confusing data. Another method of position estimation is the calculation of the instantaneous center of rotation (COR) of the target using all three markers (40). In a rigid body the COR is unambiguous avoiding the confusion created by the mean position option. It also removes the influence of rotation on the position estimate, i.e., markers can translate during pure target rotations (41). With only three markers COR is a less robust solution; however, with additional redundant markers COR can be calculated from any set of three markers protecting from data loss as a result of occlusion. In fact with more than three available markers the most representative COR can be selected or solved by least squares giving the system an inherent robustness to noise and outlying estimates (42). Target orientation estimation would similarly benefit from the addition of redundant markers as a best fit solution could be chosen from multiple three marker estimates. However, in this application COR does have some important flaws: in the presence of small rotations, as is the case in static posturography, COR is extremely susceptible to noise (43); in this case we also expect COR to simply correspond the position of the subject's ankles and not their COM since, assuming they have adopted an ankle strategy, this is the position they are rotating about. For these reasons COR was not used for this application. Multiple markers were also determined to be unnecessary as they were rarely occluded, nevertheless in more challenging environments they would be a good addition.

It has been shown that during quiet standing tasks, points affixed to a subject's body have proportional movement (16). Considering a subject's COM as such a point it is expected that stereo vision measured TP would be proportional to COM when the target is affixed to the subject's shoulder. Our *in vivo* results indicate that this is the case. In the quiet standing task COP must oscillate about COM to maintain balance (3); our data demonstrated COP oscillation about TP. In gross movements COP must shift in the direction of COM movement to counteract the body's momentum; our data demonstrated this with a good positive correlation between COP and TP in both the *x* and *y* coordinates (Spearman's, ρ ≥ 0.73). Finally COM is composed of frequencies below 1 Hz since the human body acts as a mechanical low pass filter (44); we measured the TP's power spectrum which matched this frequency range (f99 < 1.1 Hz). Stereo vision measured TP can therefore be used as a reasonable estimate of scaled COM in quiet standing tasks when stability is maintained.

Both stereo vision and force plate systems were able to detect the increased task difficulty between the eyes open and the eyes closed conditions. This challenge was demonstrated by the order of magnitude increase in traditional posturographic parameters: path length, and position range. However, a more complete analysis of the subject's postural control could be determined by considering the data captured by both systems in complement. During the easier eyes open condition, COP (captured by the force plate) simply oscillated about TP (captured by the stereo vision system) which indicated that the subject was able to maintain balance by using an ankle strategy. In contrast the harder eyes closed condition necessitated a combined hip-ankle strategy after the subject became unstable. Having reached their limit of stability (as identified by the force plate) the subject pivoted at their hips (as identified by the stereo vision system) to maintain stability. This insight into employed strategy could not be identified without the data from both the force plate and the stereo vision system. Further parametric analysis could therefore be based on adopted strategy rather than some assumed strategy.

This study presents preliminary results for the developed stereo vision system and its use in complement with a force plate. In order to validate the use of this system in a clinical setting further work is required to standardize the interpretation of the measured sway including identification of postural strategies, and parameterization of COM control for balance assessment. Future work must also establish normal reference values across gender and age in a healthy population followed by an analysis of deviation from these reference values given populations which different pathologies. Correlation was shown between the developed stereo vision system and a force plate in static posturography. This stereo vision system can be used to measure sway while subjects stand on a platform with a foam surface or a moving platform. With this configuration the contribution of proprioception and vision to the vestibulo-spinal response can be minimized to isolate the vestibular contribution. COP/sway and latency to step during a balance perturbation, via the platform or directly to the body, also provide functional test measures of vestibular function. For further clinical validation future work should correlate these measures with other established optical systems followed by other technologies including dynamic force platforms, inertial sensors, and electromagnetic trackers.

## Conclusions

A stereo vision system was developed to directly measure 6D human sway for static posturography. This approach was inexpensive and made accessible by the abundant resources available for stereo vision development. Preliminary results show 3D position and orientation measures were precise to, at worst, 0.1 ± 0.04 mm and 0.15 ± 0.06°, respectively. Computer modeling was able to predict this precision with 11.3% mean error. 3D position and orientation measures were typically accurate to 1.1 mm and 0.8° in the worst case coordinates: depth and pitch. *In vivo* comparison between stereo vision measured position and force plate measured COP demonstrated good correlation and both systems were able to discern task difficulty. However, when used in complement, balance strategy could be identified which could inform further parametric analysis. Balance strategy could not be identified with the data from only one system. This stereo vision system coupled to a foam platform or a balance perturbation system can be used to provide a functional test of vestibular function. For clinical use, future work must standardize balance assessment parameters, establish normal reference values across gender and age in healthy and pathologic populations, and investigate correlation with existing systems.

## Ethics statement

This study was carried out in accordance with the recommendations of the National Statement on Ethical Conduct in Human Research and processes outlined in the UNSW Human Research Ethics Operations Manual, UNSW Human Ethics Committee. The protocol was approved by the UNSW Human Ethics Committee. All subjects gave written informed consent in accordance with the Declaration of Helsinki.

## Author contributions

AM conceived and supervised this work. WF carried out the research, modeling, implementation, validation, and analysis with input from AM. WF wrote the manuscript with critical feedback from AM.

### Conflict of interest statement

The authors declare that the research was conducted in the absence of any commercial or financial relationships that could be construed as a potential conflict of interest.
